# Tophi and frequent gout flares are associated with impairments to quality of life, productivity, and increased healthcare resource use: Results from a cross-sectional survey

**DOI:** 10.1186/1477-7525-10-117

**Published:** 2012-09-22

**Authors:** Puja P Khanna, George Nuki, Thomas Bardin, Anne-Kathrin Tausche, Anna Forsythe, Amir Goren, Jeffrey Vietri, Dinesh Khanna

**Affiliations:** 1 University of Michigan, Michigan, USA; 2 Western General Hospital, Edinburgh, UK; 3 Hospital Lariboisiere, Paris, France; 4 University Clinic, Dresden, Germany; 5 Novartis Pharmaceuticals, Inc, East Hanover, NJ, USA; 6 Kantar Health, New York, NY, USA; 7 Kantar Health, Princeton, NJ, USA

**Keywords:** Gout, Quality of life, Productivity, Refractory chronic gout

## Abstract

**Background:**

The prevalence of gout is increasing, and most research on the associated burden has focused on serum urate (sUA) levels. The present study quantifies the impact of the presence of tophi and frequency of acute gout attacks on health-related quality of life (HRQOL), productivity, and healthcare resource utilization.

**Methods:**

Patients with self-reported gout (n = 620; 338 in US and 282 across France, Germany, and UK) were contacted based on inclusion in the 2010 US and EU National Health and Wellness Surveys (Kantar Health) and the Lightspeed Research ailment panel. Respondents were categorized into mutually-exclusive groups based on number of gout flares experienced in the past 12 months (0/don’t recall, 1–2, 3, 4–5, 6+), current presence of tophi (none, 1+, or not sure), and sUA level awareness (yes, no). HRQOL (SF-12v2), healthcare provider visits in the last 6 months, and work productivity and activity impairment (WPAI) were compared across groups.

**Results:**

Most patients were males, mean age of 61 years, who reported experiencing at least one acute gout flare in the past 12 months, and 12.3% (n = 76) reported presence of tophi. Among the 27.7% (n = 172) of patients who were aware of their sUA levels, higher sUA was associated with more flares and tophi. Decreased HRQOL was associated with more frequent flares and presence of tophi. In multivariable models predicting outcomes based on presence of tophi and number of flares, both flares (≥4) and tophi (≥1) were associated with HRQOL decrements on physical and mental component summary scores and health utilities (all *p* < 0.05), after adjustment for age, gender, and time since diagnosis. Flares were also associated with greater activity impairment.

**Conclusions:**

Impairments associated with gout flares and presence of tophi, across patients in the US and EU, underscore the importance of effective management of this potentially curable condition.

## Background

Gout affects 3.9% of the adult population in the US (8.3 million) [1] and over 1% of the adult population in Germany and the UK [2]. Gout has increased in prevalence worldwide [3,4] and it is the most common inflammatory arthritis in men [5]. Acute gout manifests when monosodium urate (MSU) crystallizes and deposits in joints, bursae or tendon sheaths, and provokes an inflammatory response that causes a typical gout flare. This flare is characterized by an acute onset (maximum within 24 hours) of a heavily inflamed and extremely painful mono- or oligoarthritis which often results in short-term sick leave [6]. Serum urate (sUA) concentration above the limit of solubility (>6.8 mg/dL/400 μmol/L) leads to crystal deposition which is a necessary precursor for this disease, though many with hyperuricemia will never develop gout [7,8]. Gout patients whose sUA is maintained below 6 mg/dL (360 μmol/L) over time can expect to remain flare free and this is a most important aspect of long-term management of gout [9-12]. Higher sUA levels predict more flares and development of tophi [2,13-16], and maintaining lower sUA prevents the formation of new tophi and reduces the size of established ones [17,18].

Gout patients frequently have a number of comorbidities, including obesity, hypertension, high serum lipid and cholesterol levels, kidney disease, diabetes, and cardiovascular disease [2,7,19-21]. Due to the rising incidence and prevalence of gout, greater scrutiny has been directed towards the impact of gout on health-related quality of life (HRQOL) [22,23], healthcare resource utilization, and work productivity, a task complicated by the presence of the other ailments.

Although there is an expanding literature on the humanistic and economic burden of gout, gaps remain. Much of the current literature linking gout to HRQOL either relies on data from US veterans [24], patients at a small number of medical facilities [20], or studies with small sample sizes [22], while data on work productivity and activity impairment in gout is very sparse.

The objective of the present study was to better understand the burden associated with signs and symptoms of gout (namely, tophi and flares) in larger, more diverse, and more representative samples of patients across the US and EU than has been reported previously. The secondary objective was to distinguish the burden of symptoms directly attributable to gout from those associated with comorbid diseases. By highlighting the heterogeneity of disease burden within the gout-diagnosed population, examining the impact of both tophi and flares across samples of US and EU patients, this study adds breadth and specificity to current analyses of gout burden in the literature and can thus contribute to more effective disease management and improved patient outcomes.

## Methods

### Data source

Self-reported data were obtained from patients identified through the US and EU versions of the 2010 National Health and Wellness Survey (NHWS; Kantar Health, New York, NY, USA) and the Lightspeed Research (LSR; New York, NY, USA) ailment panel (used to supplement respondents not available through NHWS). The NHWS is a demographically representative, annual cross-sectional survey of adult respondents (18 years of age and over), providing self-reported information on treatment, healthcare attitudes and behaviors, patient disease and demographic characteristics, and health-related outcomes. NHWS and LSR ailment panel members are both sourced from a more general LSR panel, whose members are recruited through opt-in emails, co-registration with panel partners, e-newsletter campaigns, online banner placements, and both internal and external affiliate networks. All panelists explicitly agreed to become panel members, registered through unique email addresses, and completed in-depth demographic registration profiles.

A stratified random sample procedure is implemented for NHWS so that the final sample mimics the demographic composition of the country in which it is administered, in order to achieve better representativeness. The US sample is stratified by age, gender, and ethnicity, and the EU sample is stratified by age and gender. Comparisons between the NHWS sample, the US census, and other national surveys have been made elsewhere [25,26].

All respondents, from both NHWS and the current study, gave informed consent, and the study was approved by the Essex Institutional Review Board (Lebanon, NJ, USA).

### Study population

Panel members reporting a physician diagnosis of gout were invited to participate via email in an online, self-administered survey. Out of 1936 patients reporting a physician diagnosis of gout invited to participate, 747 responded (a 39% response rate), and 620 patients completed the survey (563 via NHWS), including 338 (54.5%) from the US, 181 (29.2%) from the UK, 85 (13.7%) from Germany, and 16 from France (2.6%).

### Measures

Participants completed a web-based questionnaire that included questions regarding the patient’s gout and several validated scales.

#### Gout characteristics

Information was collected concerning the patient’s gout including their most recent serum urate level (sUA: <6 mg/dL/360 μmol/L, 6 - 8 mg/dL/360-480 μmol/L, or >8 mg/dL/480 μmol/L), the number of flares in the past 12 months (don’t recall, 0, 1–2, 3, 4–5, or 6+), the presence of tophi (not sure, 0, 1, or 2+), and the number of years since they were diagnosed with gout.

#### Health-related quality of life

HRQOL was assessed using the Medical Outcomes Short Form 12 (SF-12v2) questionnaire [27]. This instrument allows for the calculation of physical (PCS) and mental (MCS) component summary scores. Scores for the PCS and MCS are normed to the US population (Mean = 50, SD = 10), with higher scores indicating greater HRQOL. SF-6D health utilities were also calculated from responses to the SF-12v2 [28]. Scores for the SF-6D range from 0.29 (extremely poor health) to 1 (perfect health). Differences in PCS and MCS exceeding 3 points are considered minimally important differences (MIDs) [29], and 0.03 is the MID for the SF-6D [30].

#### Resource use

The number of visits to a traditional healthcare provider in the past six months was assessed.

#### Work productivity and activity impairment

Work productivity impairments and impairment in daily activities were assessed using the validated Work Productivity and Activity Impairment (WPAI) questionnaire [31]. Four subscales (absenteeism, presenteeism, overall work impairment, and activity impairment) were generated in the form of percentages, with higher values indicating greater impairment. *Absenteeism* represents the percentage of work time missed due to health in the past seven days. *Presenteeism* represents the percentage of impairment while at work due to health in the past seven days. *Overall work impairment* (OWI) is the total percentage of work time missed in the last 7 days due to either absenteeism or presenteeism. *Activity impairment* represents the percentage of impairment during daily activities. Only employed respondents provided data on absenteeism, presenteeism, and overall work impairment, but all respondents provided data on activity impairment.

### Statistical analyses

Descriptive statistics and frequency distributions were undertaken for all variables. Bivariate comparisons on continuous variables and scales were conducted using t-tests for comparisons between two groups and ANOVA for comparisons with three groups or more, followed by Tukey HSD *post hoc* tests. Chi-square tests were conducted in the case of categorical variables. Because symptom levels were collected as categories rather than exact numbers, correlations between levels of flares, tophi, and sUA were conducted using Spearman’s rho.

For the purposes of analysis, patients who did not know whether they had experienced flares were combined into the zero flares group, the assumption being that any flare that they might have experienced was not serious enough to have been identified by a physician. Moreover, analysis of the prevalence of flares revealed that there were only eight patients (1%) who did not recall whether they had flares. Nearly one-third (n = 195, 31%) of respondents were unsure of whether they had tophi, possibly indicating that they suspected a tophus but were not certain given the description in the questionnaire (“Tophi are deposits of crystallized uric acid that can appear as moveable lumps or whitish nodules”). Given the substantial number of such patients, it was deemed prudent to examine them as a separate group.

Multivariable models predicting healthcare resource use, work productivity, and HRQOL included frequency of flares in the last 12 months (1–2, 3, 4–5, or 6+, relative to 0/don’t recall flares), the presence of tophi (1+, or not sure, relative to 0 tophi), age, gender, and length of gout diagnosis (in years) as predictors. HRQOL analyses (MCS, PCS, and SF-6D) were conducted using maximum likelihood multiple regression to adjust the MCS and PCS scores and SF-6D health utilities for the effects of age, gender, and length of illness. Distributions of scores were examined and were normally distributed, so no transformation was applied. Because work productivity, impairment, and resource utilization are often highly skewed, we used a generalized linear model (GLM) approach specifying a negative binomial distribution. Adjusted logarithmically transformed counts were modeled in the GLM. This approach tested whether the adjusted transformed counts differed among the groups while accounting for covariates. In order to make the results more interpretable, we calculated the antilog of regression estimates, which yields rate ratio (RR) values. The rate ratio indicates the number of times greater impairment was for the given group compared with the reference group (e.g. a rate ratio value of 1.5 would indicate that the mean level of absenteeism for the ≥ 3 flares/year group is 1.5 times that of no flares).

The gout questionnaire did not assess respondents’ comorbid status. However, given the likely contribution of higher comorbidities to poorer health outcomes among respondents, comorbid status may be confounded with gout flares and tophi in the analyses. Therefore, a supplementary set of analyses was conducted on the subsample of respondents re-contacted via the NHWS (n = 563), using the same predictors and outcomes as described above, but adjusting additionally for relevant variables available from the NHWS, in which respondents had participated previously. In particular, the supplementary models controlled for self-reported diagnosis with diabetes mellitus, hypertension, or chronic kidney disease, as well as BMI category (overweight, obese, or missing BMI, vs. normal/underweight as reference). Given the loss of statistical power associated with the reduced sample, additional covariates, and noise introduced by non-contemporaneous measures assessed at different lags over the course of the previous year, the supplementary results are reported only as a footnote to the main analyses, for the purpose of helping rule out obvious confounds.

Statistical significance was set at *p* < 0.05 for all hypotheses tested. Analyses were performed using SPSS version 19.0.

## Results

### Demographics & patient characteristics

The characteristics of the sample are presented in Table 1 and Table 2. The average age was 60.9 years, and patients had been diagnosed an average of 12 years. Most were male (81%), and 38% were employed. Approximately half were on allopurinol (51%). Thirteen percent had at least one tophus, and an additional 31% were unsure of whether they had any tophi. One quarter (25%) of the sample had been flare-free for the past 12 months, while 38% experienced at least 3 flares in the same time period.

**Table 1:** Patient characteristics

	**Total sample**	
	**(n = 620)**	
	***M***	***SD***
Age, mean years (SD)	60.9	11.6
Duration of gout, years	12.0	10.7
	***n***	**%**
Male	504	81%
Employed	234	38%
sUA level		
Unknown	448	72%
<6 mg/dL/360 μmol/L	62	10%
6-8 mg/dL/360-480 μmol/L	75	12%
>8 mg/dL/480 μmol/L	35	6%
Current medications		
Allopurinol	319	51%
Febuxostat	29	5%
Probenecid	18	3%
Benzbromarone	4	1%
Other	97	16%
None	194	31%
Flares in last 12 months		
Don’t recall	8	1%
0	143	23%
1-2	235	38%
3	91	15%
4-5	86	14%
6+	57	9%
Tophi		
Don’t know	195	31%
0	349	56%
1	29	5%
2+	47	8%

**Table 2:** Patient characteristics by tophi and frequency of gout flares

	**Number of self-reported tophi**						**Number of self-report flares in past 12 months**									
	**No tophi (n =349)**		**Not sure (n = 195)**		**Any tophi (n = 76)**		**0/don’t recall (n = 151)**		**1 to 2 (n = 235)**		**3 (n = 91)**		**4 to 5 (n = 86)**		**6+ (n = 57)**	
	***n***	***%***	***n***	***%***	***n***	***%***	***n***	***%***	***n***	***%***	***n***	***%***	***n***	***%***	***n***	***%***
Male	293	84%	154	79%	57	75.0%	127	84%	202	86%	67	74%	66	77%	42	74%
Age *M* (*SD*)	61.6	(11.2)	61.1	(11.6)	57.0	(12.6)	62.0	(11.2)	61.3	(11.2)	59.9	(12.9)	59.1	(11.9)	60.5	(11.1)
Length of illness (years) *M* (SD)	11.9	(10.4)	11.6	(11.4)	13.3	(10.0)	13.5	(12.0)	12.1	(10.1)	10.6	(10.5)	9.6	(7.6)	13.5	(12.7)
Employed	140	40.1%	69	35.4%	30	39.5%	54	35.8%	94	40.0%	38	41.8%	29	33.7%	24	42.1%
Medication																
Allopurinol	182	52.1%	94	48.2%	43	56.6%	91	60.3%	104	44.3%	46	50.5%	48	55.8%	30	52.6%
Febuxostat	8	2.3%	5	2.6%	16	21.1%	4	2.6%	11	4.7%	5	5.5%	4	4.7%	5	8.8%
Probenecid	8	2.3%	4	2.1%	6	7.9%	6	4%	6	2.6%	2	2.2%	2	2.3%	2	3.5%
Benzbromarone	0	0%	0	0%	4	5.3%	0	0%	1	0.4%	1	1.1%	2	2.3%	0	0%
Other	45	12.9%	40	20.5%	12	15.8%	5	3.3%	32	13.6%	74	18.7%	26	30.2%	17	29.8%
none	120	34.4	63	32.3	11	14.5%	48	31.8%	90	38.3%	27	29.7%	17	19.8%	12	21.1%
sUA																
Unknown	253	72.5%	152	77.9%	43	56.6%	116	76.8%	168	71.5%	65	71.4%	57	66.3%	42	73.7%
<6 mg/d	45	12.9%	13	6.7%	4	5.3%	23	15.2%	28	11.9%	4	4.4%	5	5.8%	2	3.5%
6-8 mg/dL	40	11.5%	19	9.7%	16	21.1%	6	4%	30	12.8%	18	19.8%	15	17.4%	6	10.5%
>8 mg/dL	11	3.2%	11	5.6%	13	17.1%	6	4%	9	3.8%	4	4.4%	9	10.5%	7	12.3%
Tophi																
No tophi	-	-	-	-	-	-	109	72.2%	142	60.4%	44	48.4%	33	38.4%	21	36.8%
Not sure	-	-	-	-	-	-	38	25.2%	65	27.7%	33	36.3%	34	39.5%	25	43.9%
Any tophi	-	-	-	-	-	-	4	2.6%	28	11.9%	14	15.4%	19	22.1%	11	19.3%
Flares																
0/don’t recall	109	31.2%	38	19.5%	4	5.3%	-	-	-	-	-	-	-	-	-	-
1 to 2	142	40.7%	65	33.3%	28	36.8%	-	-	-	-	-	-	-	-	-	-
3	44	12.6%	33	16.9%	14	18.4%	-	-	-	-	-	-	-	-	-	-
4 to 5	33	9.5%	34	17.4%	19	25.0%	-	-	-	-	-	-	-	-	-	-
6+	21	6.0%	25	12.8%	11	14.5%	-	-	-	-	-	-	-	-	-	-

The subsample of 563 respondents sourced from the NHWS was similar to the overall sample. Mean age was 61.2 years, 82% were male, and mean duration of gout was 12 years. The distribution of flares in the past 12 months was the same as in the full sample, and the proportion of patients reporting tophi was similar (12% reporting at least 1 tophus, 33% unsure, and 55% reporting none). Information about BMI and comorbidities were also available for this subgroup. Most were obese (53.8%) or overweight (33.9%), with 9.9% underweight or of normal weight, and 2.3% declining to answer questions about weight or height. In terms of comorbidities, 5% reported chronic kidney disease, 26% reported type 2 diabetes, and 63% reported hypertension.

### sUA levels

Nearly three quarters of the patients (72%) did not know their most recent sUA level; of those who knew their levels (n = 172), 62 (36%) reported sUA below 6 mg/dL/360/μmol/L. sUA level was positively correlated with flares experienced in the last 12 months, *r*_*s*_ = 0.36, as well as the number of tophi currently present, *r*_*s*_ = 0.37, both *p* < 0.001. Tophi were also associated with a higher number of flares in the last 12 months, *r*_*s*_ = 0.29, *p* < 0.001. Being unsure of tophi was also associated with higher sUA, *r*_*s*_ = 0.19, *p* < 0.05, as well as experiencing more frequent flares, *r*_*s*_ = 0.20, *p* < 0.001.

### The burden of tophi

Tophi were associated with considerable impairment of HRQOL. Patients with tophi had lower MCS, PCS, and SF-6D scores than patients without tophi, while the scores of those patients unsure of tophi fell in-between the two other groups. *Post hoc* tests revealed that both the tophi group (≥ 1 tophus) and the unsure group had significantly impaired HRQOL relative to the no tophi group for all three measures (all *p* < 0.01). The magnitude of these decrements for all three measures exceeded the commonly accepted MIDs [29,30,32]. Mean values and omnibus tests of statistical significance are presented in Table 3*.*

**Table 3:** Health-related quality of life, work productivity, and resource use by the presence of tophi (unadjusted)

	**Number of self-reported tophi**						
	**No tophi (n = 349)**		**Not sure (n = 195)**		**Any tophi (n = 76)**		
	***M***	***SD***	***M***	***SD***	***M***	***SD***	***p***
HRQOL							
SF-12 MCS	50.07	*9.42*	46.06	*12.12*	44.44	*12.88*	<0.001
SF-12 PCS	42.62	*12.30*	38.26	*11.33*	36.90	*11.71*	<0.001
SF-6D Utility	0.73	*0.14*	0.66	*0.14*	0.64	*0.16*	<0.001
Work Productivity							
% Work missed	4.76	*17.68*	5.28	*18.09*	8.80	*13.65*	0.513
% Impairment at work	16.62	*23.42*	21.36	*24.92*	37.67	*32.13*	<0.001
% Overall work impairment	19.43	*27.59*	24.62	*28.36*	40.17	*34.51*	0.002
% Activity impairment	33.35	*32.30*	44.05	*32.08*	48.68	*34.77*	<0.001
Resource Use							
Number of traditional healthcare provider visits	5.71	*6.91*	7.02	*7.52*	7.34	*9.26*	0.065

Note: MCS: Mental Component Summary; PCS: Physical Component Summary; ER: Emergency Room.

Patients with tophi also had significantly greater impairment of work productivity and activity. Tophaceous gout was associated with greater absenteeism and overall work impairment than both the group without tophi and the unsure group (both *p* < 0.05). The presence of tophi was also associated with significantly greater impairment in daily activities as compared with those free of tophi and those unsure of their presence (both *p* < 0.01).

### The burden of flares

Unadjusted comparisons revealed that HRQOL varied across the different flare groups, with more flares associated with lower HRQOL in a dose-dependent manner. SF-12 MCS, PCS, and SF-6D health utilities were all statistically significantly lower in patients with more frequent flares (all *p* < 0.001). Although flares were associated with an apparent numerical increase in work impairment, this difference did not reach statistical significance within the employed subsample. However, more frequent flares were associated with statistically significantly greater impairment of activity in the total sample, with those having the most frequent flares suffering nearly double the activity impairment of those without. Means and tests of significance are presented in Table 4.

**Table 4:** Health-related quality of life, work productivity, and resource use by annual gout flares (unadjusted)

	**Number of self-reported flares in past 12 months**										
	**0/don't recall (n = 151)**		**1 to 2 (n = 235)**		**3 (n = 91)**		**4 to 5 (n = 86)**		**6+ (n = 57)**		
	***M***	***SD***	***M***	***SD***	***M***	***SD***	***M***	***SD***	***M***	***SD***	***p***
HRQOL											
SF-12 MCS	49.91	*9.67*	49.23	*10.30*	47.85	*10.03*	44.66	*13.03*	44.46	*13.43*	<0.0001
SF-12 PCS	43.25	*11.61*	42.72	*12.27*	38.16	*11.85*	37.04	*11.40*	33.49	*9.99*	<0.0001
SF-6D Utility	0.73	*0.12*	0.72	*0.14*	0.67	*0.15*	0.64	*0.17*	0.61	*0.13*	<0.0001
Work Productivity											
% Work missed (n = 234)	6.76	*23.15*	5.16	*16.23*	4.66	*17.42*	1.85	*5.11*	8.71	*14.99*	0.661
% Impairment at work (n = 229)	14.12	*18.35*	21.22	*26.72*	21.62	*27.84*	19.26	*26.01*	33.33	*30.60*	0.056
% Overall work impairment (n = 234)	19.80	*26.90*	23.23	*29.80*	24.29	*30.42*	19.93	*26.70*	36.58	*33.32*	0.197
% Activity impairment	28.68	*28.42*	36.77	*33.39*	43.41	*34.42*	45.58	*34.39*	54.21	*30.29*	<0.0001
Resource Use											
Number of traditional healthcare provider visits	6.91	*8.45*	5.68	*6.01*	8.29	*10.09*	8.02	*8.76*	7.40	*6.79*	0.032

Note: All n = 620 unless otherwise noted. MCS: Mental Component Summary; PCS: Physical Component Summary; ER: Emergency Room.

### Multivariable models

**Figure 1:**
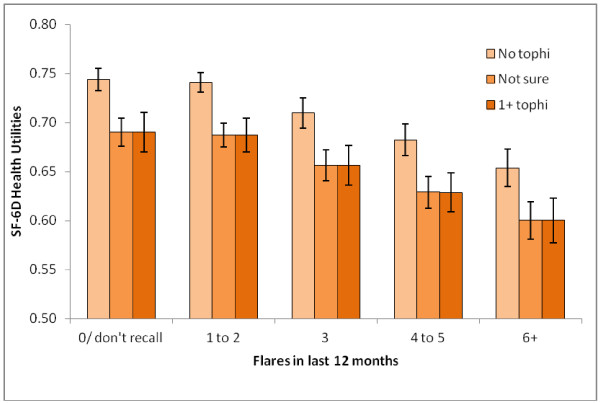
**Mean SF-6D health utilities by flare frequency and the presence of tophi, adjusted for age, gender, and length of illness.** Lower values indicate worse health; error bars represent standard error of the mean. Tophi (1+ or not sure) and flares (4+) are both associated with lower health utilities relative to no tophi and no flares/don’t recall, respectively (all *p < 0.05).*

Multivariable models, adjusting for covariates and simultaneous presence of flares and tophi, showed that tophi (vs. no tophi) were associated with statistically significantly lower MCS (*p* < 0.01) and PCS (*p* < 0.01) scores, as was being unsure of the presence of tophi (both *p* < 0.01). More frequent flares (≥ 4) were also associated with lower MCS (*p* < 0.05); those with 3 or more flares also had lower PCS scores (*p* < 0.05). The adjusted means are presented in Table 5. A similar pattern emerged in the analyses of health utilities, presented in Figure 1. As in the analysis of MCS and PCS scores, both confirmed tophi and being unsure of tophi were associated with significant impairment of HRQOL (both *p* < 0.01). More frequent flares (≥ 3) were associated with additional decrements in SF-6D utilities, after adjustment for age, gender, and length of diagnosis (*p* = 0.001).

**Table 5:** Mean MCS and PCS values by flare frequency and the presence of tophi, adjusted for age, gender, and length of illness

	**Number of self-reported flares in past 12 months**				
	**0/don't recall**	**1 to 2**	**3**	**4 to 5**	**6+**
SF-12 MCS					
No tophi	50.44	50.32	50.07	47.35	46.84
Not sure	47.20	47.07	46.83	44.11	43.59
1+ tophi	46.86	46.73	46.49	43.77	43.25
SF-12 PCS					
No tophi	44.05	43.72	40.26	39.17	36.08
Not sure	41.06	40.73	37.27	36.17	33.09
1+ tophi	40.20	39.87	36.40	35.31	32.23

Note: MCS: Mental Component Summary; PCS: Physical Component Summary.

**Figure 2:**
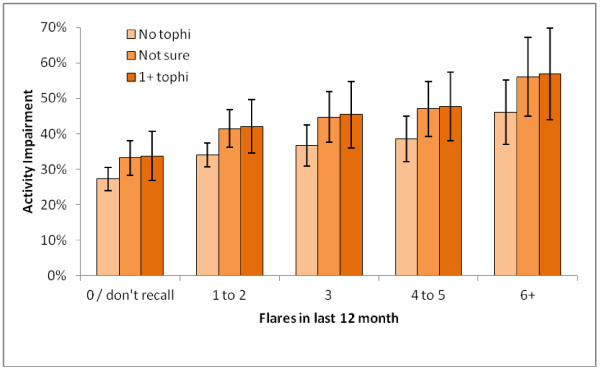
**Mean overall activity impairment by flare frequency and presence of tophi, after adjusting for age, gender, and length of illness.** Higher values indicate greater impairment; error bars represent standard error of the mean. Those with any flares are significantly more impaired than those without flares after adjusting for age, gender, and length of illness. Compared with those without tophi, those unsure of tophi were also more impaired (*p* < 0.05) and there was a trend for greater impairment among those with tophi (*p* = 0.07).

Patients with 1–2 flares in the past year showed significant activity impairment compared with those without, even after adjustment for covariates (*p* < 0.05), and impairment increased with frequency of flares. Those unsure of tophi were significantly more impaired in non-work activities than those without (*p* < 0.05), though lack of power prevented this effect from reaching conventional statistical significance among those with tophi (*p* = 0.07). The adjusted means are displayed in Figure 2.

No associations between flares or tophi and measures of work productivity were observed after adjusting for covariates, but numbers were small as these analyses were of necessity limited to the employed 38% of the sample.

We also examined the data from the smaller sample of respondents re-contacted from NHWS, and adjusting for additional covariates (i.e., BMI, diagnosis with chronic kidney disease, type 2 diabetes, and hypertension), the presence of tophi, uncertainty about tophi, and frequent flares (≥ 4) were all significantly associated with decreased MCS, PCS, and health utilities, as well as increased activity impairment. Specifically, having more than 4 flares in the past year (vs. 0/unknown) was associated with lower MCS (*p* < 0.05), PCS (*p* < 0.05), SF-6D (*p* < 0.05), and activity impairment (*p* < 0.05) in the supplementary regressions. Likewise, those who were not sure of whether they had tophi exhibited lower MCS (*p* < 0.01), PCS (*p* < 0.001), SF-6D (*p* < 0.001), and activity impairment (*p* < 0.01) than those with no tophi. Those reporting at least one tophus (vs. no tophi) also demonstrated lower MCS (*p* < 0.05), PCS (*p* < 0.01), SF-6D (*p* < 0.01), and activity impairment (p < 0.05). Despite accounting for potential confounders and with reduced power, almost all results were replicated in terms of magnitude, direction, and significance. The only discrepancies in results were that 3 flares became non-significantly associated with a health utilities decrement (*p* = 0.31), and the associations between 1–3 flares and greater activity impairment (relative to no flares) fell below conventional levels of significance (1–2 flares: *p* = 0.051; 3 flares: *p* = 0.075).

### Comparisons across the spectrum of disease phenotypes

Patients were categorized into three subgroups based on frequency of flares and the presence of tophi, without regard to treatment; 1) asymptomatic gout (no flares in the past year, no tophi), 2) severe tophaceous gout (defined as confirmed tophi and ≥ 3 flares in the past year) and 3) very severe tophaceous gout (defined as confirmed tophi and ≥ 6 flares in the past year). The groupings were similar to those employed in a previous study [33]. Both the severe group (n = 44) and very severe subgroup (n = 11) had significantly lower SF-6D health utilities than did the asymptomatic group (both *p* < 0.01).

**Figure 3:**
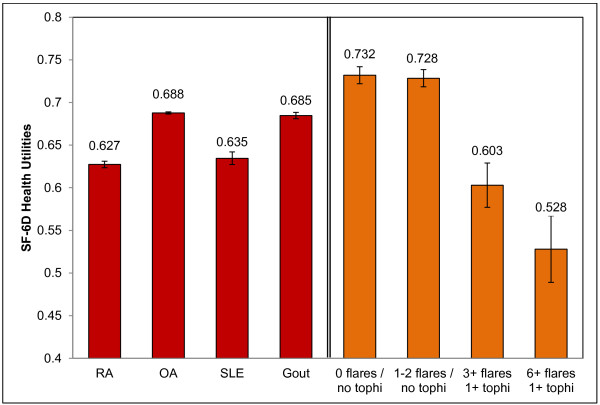
**Average health utilities of patients with gout and patients with other rheumatic diseases.** Average health utilities are unadjusted values taken from 2010 US NHWS (red) and the current study (orange). Error bars represent standard error of the mean. Mean utility for gout with 6+ flares and tophi is significantly worse than all comparison conditions, *p* < 0.05. RA: rheumatoid arthritis; OA: osteoarthritis; SLE: systemic lupus erythematosus.

The average SF-6D scores of the severe and very severe gout patients were also compared with the average SF-6D scores of US patients with other chronic rheumatic diseases. Average SF-6D scores for gout patients who did not report rheumatoid arthritis (RA), osteoarthritis (OA), or systemic lupus erythematosus (SLE), and average utilities of patients with RA, OA, and SLE patients without comorbid gout were calculated from the 2010 US NHWS. The means are presented in Figure 3. Independent-sample t-tests revealed that asymptomatic gout patients had higher health utilities than all other patient groups (all *p* < 0.05). In contrast to the asymptomatic patients, those in our sample with severe gout had health utilities similar to the average SLE or RA patient (both *p* > 0.05), and significantly worse than the average gout or osteoarthritis patient in the NHWS (*p* < 0.05). Patients with very severe gout had significantly lower health utilities than any comparison group (*p* < 0.01).

## Discussion

This study demonstrates that gout patients from the US and three EU countries suffer from significantly decreased HRQOL, with the average patient suffering decreased mental as well as physical well-being, relative to population norms. These decrements in HRQOL vary across the spectrum of clinical phenotypes according to the presence and frequency of gout symptoms and signs. Patients with confirmed tophi and more frequent acute gout attacks had lower HRQOL, and the association between HRQOL, tophi and flares remained significant even after accounting for covariates. Impairments were not limited to HRQOL, and unadjusted comparisons revealed that patients reporting tophi had greater work impairment than those without, while more frequent flares were associated with increased healthcare resource use. Both flares and tophi were also associated with significant impairments in non-work activities in multivariable models.

Impairment of the physical components of quality of life have been found consistently in studies of gout patients [22,24,34,35], and was notably related to the burden of symptoms and signs in this sample. Those with more severe gout had low PCS compared with both population norms and those gout patients who were free of tophi and flares. These differences were statistically significant, and greater than those which are generally accepted as being clinically meaningful. These studies confirm a relationship between symptom load and HRQOL previously observed in smaller samples of gout patients in the US [20,35]. The PCS levels in the most severely affected patients in these studies were comparable to those reported in previous studies of patients with severe symptomatic gout and patients with gout who had not responded to urate-lowering therapy [20,35].

Gout patients are known to use more healthcare resources. An analysis of claims data showed that gout sufferers incurred more costs for medical claims, prescription claims, sick leave, short-term disability, and worker’s compensation than did other employees [36]. Another database study of gout in the elderly found higher healthcare resource utilization in gout patients than in matched controls, which was attributable in part to having more comorbidities [37]. While comorbid conditions may account for some of the elevated resource use among gout patients, gout-related healthcare utilization increases with severity of gout [38]. A study using the MarketScan database showed that patients having 3 or more flares per year had more comorbidities, and incurred $10,222 more per year in healthcare costs than age and gender matched controls without gout [33]. Administrative claims data also show that higher sUA is associated not only with a greater number of flares, but also with higher costs per flare [16,39].

There is evidence that gout affects worker productivity. A diary study of patients with chronic gout refractory to conventional urate-lowering therapy found an average annual workday loss of 25 days [36]. Another study showed that employees with gout missed 4.56 more days of work per year [40]. Gout symptoms and signs were also associated with impairment in daily activities, although the limited number of patients reporting tophi restricted the possibility of detecting activity impairment associated with tophi over and above the strong effect of flares. It is important to bear in mind that these impairments are compared with those of gout patients who are free of acute symptoms, rather than those in the population at large. It seems likely that comparisons between these patients and the general population would demonstrate larger differences in HRQOL and impairment of activities. The failure to demonstrate significant reduction in work productivity in the gout patients participating in these studies should not be interpreted as indicating that gout has no effect on work performance or productivity. The number of patients in employment in the study was relatively small and as the average age was 61 years, many are likely to have been in retirement. A recent diary study documented productivity impairments due to gout flares [41], a finding that needs to be confirmed in a larger sample of employed patients. It is certainly possible, however, that patients with severe gout lose their jobs or decide to retire as a result of their disease; such loss of productivity would not be ascertained in a diary study or with the WPAI questionnaire used in this study.

In the present study, patients who reported symptoms of inadequately controlled chronic gout (similar to refractory chronic gout), defined here as at least three flares in the past year and the presence of at least one tophus, had severely impaired HRQOL, with SF-6D health utilities 0.13 below those in patients free of tophi and free of flares for the past 12 months. The magnitude of these decrements can be placed into context by comparing the health utilities of patients with gout across the spectrum of clinical phenotypes with those reported by patients with other rheumatic diseases. As indicated in Figure 3, patients with gout had similar health utilities to patients suffering from RA or SLE. Those with most severe gout, characterized by tophi and six or more flares in the past year, had health utilities that were significantly lower than the average for either RA or SLE. The findings emphasize the importance of seeking to provide effective treatment for all patients with gout and particularly those with tophi and frequent flares.

Our study has a number of limitations. As in all cross-sectional analyses based on self-reported patient information the data reported may be subject to recall bias and the ability of the patients to accurately report information about their condition. A total of 8 patients did not recall whether they had flares. While this small number was not expected to alter results significantly, to the extent that there were patients with flares in that unknown group, this would render our results more conservative. The survey did not attempt to ascertain the size or location of tophi, which may be an important determinant of impairment of HRQOL in patients with tophaceous gout. Although it is likely that comorbid status was confounded with tophi/flares in contributing to poorer health outcomes, controlling for comorbidities (and BMI) in the current study did not detract from the overall findings, in spite of the reduced statistical power. Having tophi and at least four flares in the past year were still significantly associated with the poorer outcomes. Patients’ diagnosis with gout was not verified. Previous studies have, however, shown that a high proportion of self-reported cases of gout meet classification criteria when assessed by physician, hospital discharge diagnosis, or use of gout-specific medication [20,42-44]. The generalizability of the findings may be limited by self-selection of subjects into the survey panel and/or the survey itself. The relationships between variables observed are correlations which cannot be deemed to be causal. Unmeasured variables (such as the number or severity of comorbid conditions) may explain a portion of the observed effects, although an association between physical HRQOL and gout symptoms has been observed after controlling for comorbidities in a previous study [34]. The comparisons across rheumatic diseases must be interpreted with caution. No attempt was made to control for covariates or potential confounders, which may explain most or all of the observed differences in health utilities.

## Conclusions

The impairment of HRQOL in patients with gout increases with the presence of tophi and the frequency of flares, even after adjusting for covariates. Gout symptoms are also associated with greater impairment in daily activities. Gout characterized by severe symptoms and signs (i.e., 3+ flares/year and tophi) imposes a substantial and clinically meaningful burden on the patient at least comparable to the impairment of HRQOL associated with other rheumatic diseases. These findings, across representative samples of patients in both the US and EU, highlight the different potential impacts of gouty tophi and flares and underscore the importance of effective management of this potentially curable condition.

## 

Previous presentation: American College of Rheumatology, 2011 Annual Scientific Meeting, Chicago, IL, November 5–9, 2011.

## Competing interests

Savient Pharmaceuticals, Inc. provided funding for this study. AF was an employee of Savient during its execution. AG and JV are employees of Kantar Health, who conducted the study and contributed to manuscript preparation with funding from Savient. GN, TB, A-KT, and DK received funding from Savient for consulting purposes. In addition to funding from Savient, A-KT also received funding from Berlin-Chemie Menarini and Novartis for giving lectures and serving on Advisory Boards on the topic of gout; PK has served on speakers bureaus for Takeda, and has received research funding from Savient and ARDEA; DK serves as a consultant to ARDEA and Takeda, and is co-Principal Investigator for the 2012 American College of Rheumatology Guidelines for the management of gout. GN has served as a consultant for Ardea, Ipsen, Menarini, Metabolex, Novartis, and convenes the group to revise treatment guidelines for the British Society of Rheumatology. TB has received consultancy fees from the following companies, in the field of gout: Savient, Ipsen, Menarini, Takeda, Teijin, Sobi, Ardea Biosciences, Biocryst, Novartis, and Mayoli Spindler.

## Authors’ contributions

PK, GN, TB, A-KT, AF, and DK contributed to the design of the study and the writing of the manuscript. AG contributed to the design and analysis of the study, and the writing of the manuscript. JV contributed to the analysis and writing of the manuscript. All authors have read and approved the manuscript.
